# Tools to study microbial iron homeostasis and oxidative stress: current techniques and methodological gaps

**DOI:** 10.3389/fmolb.2025.1628725

**Published:** 2025-07-30

**Authors:** Patryk Strzelecki, Dariusz Nowicki

**Affiliations:** Department of Bacterial Molecular Genetics, Faculty of Biology, University of Gdańsk, Gdańsk, Poland

**Keywords:** iron, ferrous iron, virulence, pathogen, biofilm, CAS, ferrozine, ferroptosis

## Abstract

Iron is a vital nutrient for both microbial pathogens and their eukaryotic hosts, playing essential roles in stress adaptation, symbiotic interactions, virulence expression, and chronic inflammatory diseases. This review discusses current laboratory methods for iron detection and quantification in microbial cultures, host-pathogen models, and environmental samples. Microbial pathogens have evolved sophisticated specialized transport systems, iron acquisition strategies to overcome its limitation, including siderophore production, uptake of heme and host iron-binding. These iron-scavenging systems are closely linked to the regulation of virulence traits such as adhesion, motility, toxin secretion, and biofilm formation. In ESKAPEE pathogens (*Enterococcus faecium*, *Staphylococcus aureus*, *Klebsiella pneumoniae*, *Acinetobacter baumannii*, *Pseudomonas aeruginosa*, *Enterobacter* spp. and *Escherichia coli*), iron limitation enhances biofilm development, which protects bacteria from antibiotics and immune responses and promotes persistent infections. Even worse, pathogens can also manipulate host iron metabolism, exacerbating inflammation and disease progression. Although iron is indispensable for microbial growth, excessive intracellular iron promotes reactive oxygen species generation, causing oxidative damage and ferroptosis-like cell death. Understanding the dual role of iron as both a nutrient and a toxic agent highlights its importance in infection dynamics. We provide a critical overview of existing analytical techniques and emphasize the need for careful selection of methods to improve our understanding of microbial iron metabolism, host-pathogen interactions, and to support the development of new therapeutic and environmental monitoring strategies.

## 1 Introduction

Iron is an essential nutritional element required for the growth, survival, and metabolic functions of both eukaryotic host and invading microbes. It is involved extensively in biological processes such as enzymatic reactions (e.g., ribonucleotide reductase, cytochromes), DNA synthesis and repair, electron transport chains, oxidative phosphorylation, and metabolic pathways, including nitrogen fixation and respiration (Weiss and Carver, 2018). Among metals that participate in oxidation-reduction reactions, iron is notably abundant and versatile. It is widely incorporated into proteins as part of complex structures such as heme groups, iron-sulfur clusters, and non-heme iron proteins, influencing protein activity and stability significantly (Bradley et al., 2020). In natural environments, iron predominantly exists in two oxidation states: ferrous Fe(II) and ferric Fe(III). The speciation and bioavailability of iron are strongly influenced by pH and redox conditions. Under oxygenated and neutral to alkaline conditions (pH > 6.5), Fe(III) is thermodynamically favored but exhibits extremely low solubility, significantly limiting its bioavailability to aerobic microorganisms. In contrast, under anaerobic or microaerobic conditions at acidic pH (typically <5.5), the more soluble Fe(II) form prevails. While this enhances iron availability, it poses analytical challenges due to the redox-sensitive nature of Fe(II) during sampling and detection.

Microbial iron acquisition systems play critical roles in various virulence-related features (Sheldon et al., 2016). Restricted iron conditions trigger and regulate the expression of virulence factors, which have a direct impact on microbial growth and survival. More importantly, limited iron availability within host environments has driven pathogens to develop specialized iron uptake mechanisms, including the production of siderophores, direct interaction with host iron-binding proteins (e.g., transferrin, lactoferrin), utilization of heme-binding proteins, and iron transport via specialized receptors (e.g., TonB-dependent transporters, Feo-like Fe(II) uptake system (Post et al., 2019). These mechanisms often affect other crucial virulence processes such as bacterial adhesion, motility, toxin secretion, and biofilm formation (Sheldon et al., 2016). Iron availability critically influences both antibiotic resistance and biofilm formation, a key virulence trait among ESKAPEE pathogens (*Enterococcus faecium, Staphylococcus aureus, Klebsiella pneumoniae, Acinetobacter baumannii, Pseudomonas aeruginosa, Enterobacter* spp. and *Escherichia coli*). Understanding the molecular mechanisms and regulatory networks that control microbial iron homeostasis and oxidative stress provides critical insights into bacterial pathogenesis and the broader dynamics of iron dysregulation and inflammation in host-microbe interactions. Recent studies suggest that microbial pathogens can manipulate host iron metabolism, exploiting iron to promote their survival and, in doing so, further stimulate inflammatory responses (Osterholm and Georgieff, 2015; Ganz, 2018). Therefore, the targeting of iron homeostasis has been considered as a promising therapeutic strategy (Holbein et al., 2021; Jing et al., 2024; Rosa et al., 2024; Sun et al., 2024; Zhu et al., 2025).

Biofilms are complex communities of microorganisms embedded in a self-produced extracellular polymeric matrix, significantly enhancing bacterial resistance to antibiotics, immune responses, and environmental stressors (Post et al., 2019). Under iron-limited conditions, ESKAPEE pathogens upregulate genes involved in biofilm development to facilitate more efficient iron acquisition and improve survival within hostile host environments. Iron-dependent regulators, such as Fur, modulate the expression of genes encoding biofilm-associated factors, including extracellular polysaccharides, adhesion proteins, and pili components, thus promoting bacterial aggregation and stable biofilm architecture (Oliveira et al., 2021). More importantly, recent studies highlight how interspecies interactions in polymicrobial biofilms can enhance iron acquisition and promote antibiotic resistance (Keogh et al., 2016; Govindarajan et al., 2022). In dual-species biofilms of *E. coli* and *E. faecalis*, metabolic cross-talk facilitates *E. coli* proliferation under iron scarcity via *E. faecalis*-derived L-ornithine, which induces siderophore - enterobactin - production independent of Fur regulation (Keogh et al., 2016). Moreover, it has been shown that both mono- and dual-species biofilms exhibit increased extracellular Fe(II) uptake, with *E. faecalis* dominance correlating with a tenfold increase in antibiotic tolerance. This phenotype is linked to high-affinity ferrous iron acquisition by FeoB transporters, indicating the significance of Fe(II) uptake systems in biofilm-associated antimicrobial resistance (Govindarajan et al., 2022). Enhanced biofilm formation in response to iron limitation contributes directly to chronic and persistent infections by protecting bacterial populations against antimicrobial agents and host defense mechanisms (Núñez et al., 2018).

Host organisms counter microbial infections by restricting iron availability through a strategy known as nutritional immunity (Zauberman et al., 2017; Murdoch and Skaar, 2022). This protective mechanism involves binding iron tightly with high-affinity proteins such as transferrin in blood, lactoferrin in mucosal secretions, ferritin for intracellular storage, or regulation of iron homeostasis by hormones like hepcidin (Antelo et al., 2021). Such restrictions effectively affect robust microbial expansion and virulence expression. Microbes, therefore, continually evolve more complex and diverse iron-acquisition mechanisms in response to host iron limitation (Antelo et al., 2021). In this continuous battle, some of them are able to actively adapt to limited environment (Govindarajan and Kandaswamy, 2022) by switching acquisition systems form siderophores under aerobic conditions to the ferrous Fe(II) uptake system under microaerobic conditions (Fetherston et al., 2012). As a more specialized adaptation, many bacterial pathogens have evolved strategies to acquire iron directly from host heme-containing molecules (Wang et al., 2023). Hemoproteins, such as hemoglobin, hemopexin, haptoglobin, and myoglobin, represent a major reservoir of bioavailable iron during infection. To access this pool, bacteria utilize dedicated heme and hemoglobin receptors coupled with specific transport and degradation systems (Cook-Libin et al., 2022). Heme uptake is particularly relevant during systemic infections, where extracellular hemoproteins become more abundant due to inflammation or hemolysis. However, heme excess can be toxic, hence, these systems are tightly regulated to balance iron acquisition with cellular protection.

Although iron is essential for cell homeostasis, an excess of free intracellular iron can cause oxidative stress by generating reactive oxygen species (ROS) through chemical reactions such as the Fenton and Haber–Weiss reactions (Bradley et al., 2020). Free ferrous iron reacts with hydrogen peroxide (H_2_O_2_), producing highly reactive hydroxyl radicals (·OH) and ferric iron. Ferric iron can further increase cellular ROS production and oxidative stress. Elevated ROS can damage biomolecules, resulting in DNA strand breaks, lipid peroxidation in membranes, and oxidation of proteins, leading to impaired cellular functions. This ferroptosis-like, an iron-dependent form of programmed cell death initially identified in mammalian cells and explored as a cancer treatment strategy, has recently been observed in microbial species (Cook-Libin et al., 2022; Kwun and Lee, 2023; Jing et al., 2024). Targeting microbial comprehensive regulatory systems, and detoxification mechanisms which ensure a careful balance between iron availability for critical cellular processes and protection against iron-induced damage, may provide a way to out of antimicrobial resistance crisis. This highlights iron regulation’s broader biological significance and potential therapeutic applications in infectious disease management.

In addition to its central role in host-pathogen interactions, microbial iron metabolism also contributes substantially to environmental processes through biogeochemical cycling. Many bacteria participate in iron oxidation and reduction, thereby driving essential geochemical transformations (Kappler et al., 2021). These microbially mediated redox processes affect global phenomena such as ocean productivity, carbon sequestration, and the environmental fate of contaminants (Xia et al., 2025). Alterations in iron speciation, triggered by microbial activity, influence the solubility, mobility, and bioavailability of a wide range of elements, including nutrients and toxic metals (Bonnain et al., 2016; Whitby et al., 2020). Recent advances have revealed previously unrecognized microbial pathways, such as ammonium and methane oxidation coupled to Fe(III) reduction (Kappler et al., 2021). Moreover, iron redox processes often overlap spatially and may occur simultaneously or cyclically, with microbial populations engaging in both oxidation and reduction within the same ecological niche. These findings underscore the significance of microbial iron metabolism not only in host-associated niches but also in broader ecosystems, including soils, sediments, and aquatic environments. Understanding these interactions requires accurate and context-specific iron quantification tools capable of capturing dynamic speciation and redox cycling *in situ*.

In this review, we critically examine the current state of available technics and approaches to decipher iron and its ions in microbial related specimens. We believe that reliable evaluation of iron levels allows to better understand microbial physiology, disease mechanisms, and environmental interactions. Choosing appropriate analytical methods based on specific research needs and sample types is critical for accurately understanding biological processes involving iron, developing effective treatments, and improving environmental monitoring.

## 2 How to detect and quantify iron in microbial systems

As discussed above, accurate detection and quantification of iron in microbial systems is crucial for understanding its role in metabolism, virulence regulation, and stress adaptation. However, this task poses considerable methodological challenges due to the variable oxidation states of iron, its strong tendency to form complexes with biomolecules, and its often low and fluctuating intracellular concentrations. Additionally, the presence of structurally or chemically similar metal ions or complexes in biological samples can interfere with selective iron detection. These factors necessitate the use of well-validated and often highly specific analytical techniques to ensure accurate measurement and interpretation. Below, we outline the most commonly employed methods for assessing iron in microbial samples and discuss their respective strengths and limitations (Table 1).

**TABLE 1:** The methods of iron detection and quantification.

Category	Method	Type	Description	References
Colorimetric assays	Ferrozine assay	Direct	Forms a purple complex with Fe(II), absorbance at ∼562 nm. The ferrozine assay is highly specific for Fe(II) and enables sensitive quantification in the low micromolar range.	Stookey (1970), Cowart et al. (1993), Riemer et al. (2004), Im et al. (2013)
	Phenanthroline assay	Direct	Forms an orange complex with Fe(II), absorbance at ∼510–514 nm. This assay exhibits lower specificity than the ferrozine assay and is more susceptible to interference from other metal ions and sample constituents.	Harvey et al. (1955), Komadel and Stucki (1988), Özyürek et al. (2007), Fernandes et al. (2023)
	Bathophenanthroline assay	Direct	Water-soluble derivative, forms a red complex with Fe(II), absorbance at ∼530–535 nm. Applicable for indirect measurement of chelation effects.	Lee and Stumm (1960), Perry and San Clemente (1977), Cowart et al. (1993), Freinbichler et al. (2020)
Atomic Absorption Spectroscopy (AAS)	Flame AAS	Direct	Atomization in flame to measure iron absorption.	Tautkus et al. (2004), Yaman and Kaya (2005)
	Graphite Furnace AAS	Direct	Atomization in graphite tube; higher sensitivity than flame AAS.	Kragten and Reynaert (1974), Miller-Ihli (1989), Butcher (2024)
Electrochemical methods	Voltammetry	Direct	*In situ* measurement of Fe(II) and O_2_ using redox-active electrodes Iron is deposited on electrode, then stripped while recording current potential.	Van Staden and Matoetoe (1998), Abollino et al. (2019), Borrill et al. (2019), Wygant and Lambert (2022)
	Amperometry	Direct	Measures current from Fe(II) oxidation at a working electrode and controlled potential; useful for biofilms and bacterial cultures.	Adeloju (2004), Saavedra and Cortón (2014)
	Potentiometry	Direct	Measures potential between indicator and reference electrodes, applicable to iron speciation studies and continuous monitoring in industrial or environmental systems.	Amos and Brown (1963), Bralić and Radić (1999)
Radiometric assays	Radioisotope detection	Indirect	Tracks uptake and metabolism of radiolabeled iron, most commonly using ^55^Fe or ^59^Fe isotopes, assessed by scintillation counting.	Hantke (1981), Lewis (2010)
Fluorescence and chemiluminescence methods	Calcein/Calcein-AM assay	Direct	Fluorescence is quenched by both Fe(II) and Fe(III), enabling detection of labile iron regardless of its oxidation state. The assay is sensitive to changes in total labile iron availability (e.g., in response to siderophore activity); Ex: 494 nm, Em: 517 nm.	Glickstein et al. (2005)
	Phen Green SK assay	Direct	Fluorescence quenching by labile iron ions; suitable for microbes with autofluorescence; Ex: 507 nm Em: 532 nm.	Petrat et al. (1999), 2000; Oter et al. (2007)
	Pyoverdine fluorescence monitoring	Indirect	Iron binding quenches natural siderophore pyoverdine fluorescence. Specific to *Pseudomonas* species; Ex: 360–410 Em: 450–480 nm.	Meyer (2000)
	FerroOrange assay	Direct	Cell-permeable fluorescent probe highly selective for Fe(II); enables live-cell imaging of labile ferrous iron. Ex: 543 nm, Em: 580 nm.	Yang et al. (2022), Fu et al. (2023), Grubwieser et al. (2024)
	Luminol-based chemiluminescence assay	Indirect	Light emission from reaction of luminol with H_2_O_2_ in presence of catalytic iron. Applicable in semi-quantitative estimation of the catalytic contribution of Fe(II).	Pietrzak and Denes (1996), Khan et al. (2014)
	BODIPY-CL	Direct	Highly sensitive and selective fluorochrome for Fe(III); fluorescence is quenched upon binding Fe(III), enabling detection of labile ferric iron in live cells and tissues. Suitable for cellular imaging applications. Ex: 371 nm, Em: 516 nm.	
Chromatographic and coupled methods	High-Performance Liquid Chromatography	Direct	Separates iron species; detection by UV-Vis, ICP-MS, or electrochemical means.	Şenyuva et al. (2002), McCormack et al. (2003), Boiteau et al. (2013), Proch and Niedzielski (2021)
	Ion Chromatography	Direct	Separates ionic Fe(II)/Fe(III); often coupled with post-column colorimetric or conductivity detection.	Kadurugamuwa et al. (1987), Schnell et al. (1998), Michalski (2009)
Siderophore assays	CAS assay and derivatives	Indirect	Measures iron-binding by siderophores; color change from blue to orange upon iron chelation.	Neilands (1981), Alexander and Zuberer (1991), Hider and Kong (2010), Louden et al. (2011)
Molecular biology methods	Reporter gene assays	Indirect	Monitors iron-responsive promoter activity through reporter expression.	Cotter et al. (1992), Escolar et al. (1999), Jiang et al. (2008)
	Transcriptional studies	Indirect	qRT-PCR or RNA-seq to assess mRNA levels of iron-regulated genes.	Nielsen and Boye (2005), Butcher and Stintzi (2013), Fortuna et al. (2019), Ibraim et al. (2019), Rocha et al. (2020)

### 2.1 Colorimetric assays

**FIGURE 1:**
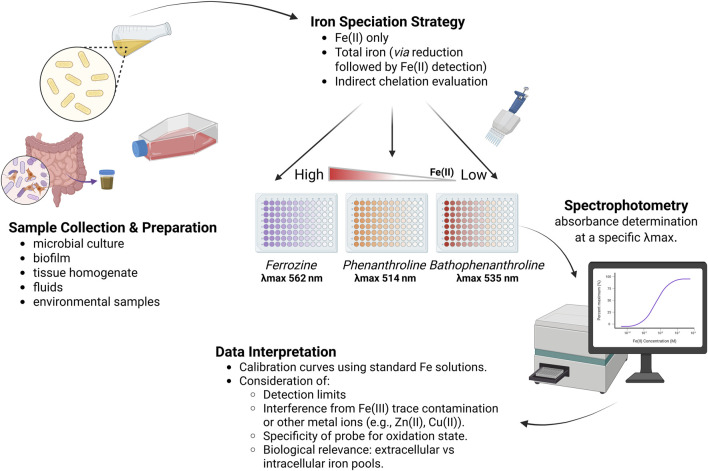
Colorimetric techniques workflow for Fe(II)/Fe(III) detection in biological samples. Samples such as microbial cultures or tissue extracts are processed and assessed for iron content using spectrophotometric probes (e.g., Ferrozine, Phenanthroline, Bathophenanthroline). Absorbance at specific wavelengths (λ_max_) reflects Fe(II) or total iron levels. Data interpretation requires calibration curves and consideration of probe specificity, detection limits, and sample context.

Colorimetric assays remain the most popular rapid and budget iron quantification option (Figure 1). Colorimetric assays use the ability of Fe(II) to create color-specific complexes with ligands (or, less often, Fe(II) with cyanides) and measure absorbance at a particular wavelength - for example, 562 nm for ferrozine, 510 nm for 1,10-phenanthroline, 533 nm for bathophenanthroline disulfonate, and 522 nm for 2,2′-bipyridyl. Spectrophotometric evaluation of Fe(III) in microbial and environmental samples remains challenging due to the relatively low direct chromogenic response of Fe(III) with common ligands and its tendency to exist in multiple oxidation states. However, ferric pools can be quantified indirectly by samples processing such as acidification or reduction. Final iron concentration can be calculated by creating a standard curve. Further in this chapter, we describe the most commonly used reagents, key steps, and advantages and limitations of each method.

#### 2.1.1 Ferrozine assay

Using Ferrozine is one of the most used methods to detect Fe(II) (Im et al., 2013). The ferrozine assay typically detects Fe(II) in the 2–1,000 µM range; however, methodological optimizations can enhance its sensitivity down to approximately 0.5 µM (Riemer et al., 2004), making it precise and cost-effective technique. Ferrozine is relatively inexpensive, and the whole procedure can be performed within 1 h, but it requires complete reduction of ferric ions (Riemer et al., 2004; Tamarit et al., 2006; Im et al., 2013). High concentrations of manganese or copper can lead to underestimated results, It is important to take that into consideration when performing the assay (Dubinsky et al., 2010; Im et al., 2013). The original paper describing this method was published in 1970 by Stookey (1970) who described it as a highly selective, intense purple complex with λ max at ∼562, and recommended 1 min incubation time (Stookey, 1970). After this, the methods have been mostly used for Fe quantification in chemical samples with slight modification, including longer incubation time (Kostka and Luther, 1994; Thamdrup et al., 1994; Riemer et al., 2004). The biological applications have been presented in the 1990s and 2000s (Riemer et al., 2004). In 2004, Riemer et al. adapted the protocol for use in eukaryotic cells, with the protocol including HCl/KMnO_4_ pretreatment to release from proteins and achieve full reduction. The redefined protocol recommends protein precipitation to avoid background interference (Riemer et al., 2004). The method has also been widely applied to study bacteria (Dubinsky et al., 2010; Akob et al., 2012; Shin et al., 2021) and yeast (Tamarit et al., 2006). Shin et al., in 2020 used ferrozine to examine the uptake of iron in *S*. *aureus.* The cells were Fe deprived by using 2-bipyridyl, and then re-exposed to FeSO_4_. Then the cells were lysed, and acidified to release ions. The final reaction mixture contained neocuproine to prevent the interference from copper. This approach enabled us to look into the intercellular activity of iron transport systems under different experimental conditions (Shin et al., 2021). In the study by Dubinsky et al. from 2010 (Dubinsky et al., 2010), ferrozine was used to measure the extracellular iron produced during microbial iron reduction. The ion were released with the use of HCL, and the supernatant was then filtered. The final ferrozine solution has been prepared in HEPES buffer. This simple procedure allowed for examining iron reduction activity in soil, showing the involvement of bacteria in biogeochemical processes (Dubinsky et al., 2010). Nevertheless, the simplicity of the approach allows to study more complex behaviors in symbiotic roots related species (Giacalone et al., 2025). Optimized ferrozine assay is proposed to measurement of redox-active metabolites (RAMs) associated with natural and agricultural systems, a class of secondary metabolites that can help bacteria solubilize phosphorus. Ferrozine assay started as a simple chemical test, and over time evolved into a commonly used method for iron detection in microbiological studies.

#### 2.1.2 Phenanthroline assay

1,10-Phenanthroline creates an orange complex with Fe(II) (Harvey et al., 1955). The working range of the dye is 0.2–100 µM and displays a strong absorbance at λ max ≈510–514 nm (Komadel and Stucki, 1988; Fernandes et al., 2023). The advantages of this technique include good repeatability and the possibility of rapid measurement in the field by using a handheld photometer (Fernandes et al., 2023). One of the first protocols was developed by Harvey et al., in 1955 (Harvey et al., 1955), allowing for both Fe(II) and total iron quantification by introducing a chemical reduction step (Harvey et al., 1955). An important feature is their photochemical behavior. Light exposure (<500 nm) can induce reduction from Fe(III) without the need for a reducing agent (Komadel and Stucki, 1988). The downside is lower sensitivity as copper, zinc, or nickel ions form weaker but still absorbent complexes, which might introduce additional steps to the reaction to mask them with agents like neocuproine [23, 26]. Although ferrozine gradually replaced phenanthroline, it is still relevant in field applications because of its easy and robust procedure (Fernandes et al., 2023). Recent technological developments have improved the utility of this method by the design of a self-contained, portable, and compact iron measurement system (IMS) based on spectroscopic absorption for the determination of Fe(II). That allows for detection limits as low as 2.5 µg Fe^2+^/L (Fernandes et al., 2023).

#### 2.1.3 Bathophenanthroline assay

Bathophenanthroline disulfonate is a sulfonated derivative of phenanthroline that is water-soluble, which makes it the most sensitive (µM range) among phenanthroline compounds (Lee and Stumm, 1960; Cowart et al., 1993; Naka et al., 2000). One of the limitations is the higher cost of the reagents. Additionally, in the presence of strong antioxidants, the background signal may be increased due to autooxidation, so samples should be measured as soon after preparation as possible (Cowart et al., 1993). One of the first papers written by Lee and Stumm in 1960 (Lee and Stumm, 1960) introduced the use of bathophenanthroline for Fe determination in water samples, under acidic conditions (pH 3–4), it forms a red complex specifically with Fe(II), without significant interference from Fe (III) (Lee and Stumm, 1960). In 1977, Perry and San Clemente (1977) addressed some limitations of this technique, especially in reducing the Fe(III) to Fe(II) before the reaction. They showed that conventional reduction with hydroxylamine was inefficient. Their approach with the use of L-ascorbic acid, HCL, and heating improved the reaction up to 82%, which significantly improved the reaction, especially for low iron samples like microbiological media (Perry and San Clemente, 1977). Freinbichler et al. (2020) extended the application further in 2019 to the *in vivo* neurochemical studies, introducing high-performance liquid chromatography using bathophenanthroline disulfonate. This allowed for the detection of Fe in very small volumes (a few microliters). This modification has been very significant for looking into iron dysregulation in neurodegenerative diseases (Freinbichler et al., 2020).

Since the introduction of the ferrozine assay, it has undergone optimization and can now be used to determine Fe concentration in different environments. Meanwhile, older methods, including phenanthroline and bathophenanthroline assays, have not been used as extensively but remain important in a specific setting, e.g., such as anaerobic systems, detection in acidic samples, or microfluidic applications. Moreover, the phenanthroline and ferrozine methods are commonly used to quantify Fe(II) in Fenton reactions, with particular importance for iron cycling and oxygenation processes. However, reaction systems can be affected by the presence of Fe(III). Namely, Fe(III) can interfere by oxidizing heterocyclic amines or by forming its own complexes, potentially leading to overestimate Fe(II) concentrations as (Yang et al., 2020). To avoid this, temperature, pH, and buffer concentration should be carefully controlled. Incubation time must be minimized and standardized across samples. Yang and colleagues proposed fluoride as a remedy of residual ferric contamination (Yang et al., 2020).

### 2.2 Atomic absorption spectroscopy (AAS)

AAS has been a widely used method for the detection of ions in a varied range of samples, including biological (Paudel et al., 2021). Its working principle is that atoms that are present in the gas phase absorb light at specific wavelengths (Putri Maharani et al., n.d.; Paudel et al., 2021). For Fe detection, the absorption is typically monitored at 248.3 nm (Ansari et al., 2008). To achieve this state, the samples must first be digested, typically with strong acid (e.g., HNO_3_, HCl, H_2_SO_4_ or their mixtures), to release iron from proteins, siderophores, or complexes. The digestion process can be enhanced by heating and ultrasonification, samples are then filtered and appropriately diluted prior to analysis. To ensure accuracy and reproducibility, measurements are performed against appropriate calibration standards, and results are quantified based on a calibration curve constructed from iron solutions of known concentrations. For microbiological samples, two variants are the most commonly used: Flame AAS (FAAS) and Graphite Furnace AAS (GFAAS).

#### 2.2.1 Flame AAS

In this variant, the sample is nebulized into a mist and aspirated into a flame, where ions get atomized. The concentration is then determined by the change in intensity of the light beam. FAAS offers several advantages, such as high throughput, moderate costs, and a relatively simple procedure. However, its limit of detection typically ranges from 2.5 to 10 μg/L, depending on matrix complexity and instrumentation (Tautkus et al., 2004; Ward and Crichton, 2015). Yaman and Kaya, in their work from 2005, present an approach to distinguishing between iron oxidation levels using solvent extraction. Their methods involve using 1-(2-Pyridylazo)-2-naphthol (PAN), which creates a complex with Fe(II)^,^ then extracting this complex using chloroform. The remaining Fe(III) requires reduction before the qualification. This technique proved to be relatively simple, selective, and sensitive (Yaman and Kaya, 2005).

#### 2.2.2 Graphite furnace atomic absorption spectroscopy (GFAAS)

Graphite Furnace AAS, also known as Electrothermal AAS, enhances sensitivity by atomizing the sample in a small graphite tube rather than in an open flame (Kragten and Reynaert, 1974; Butcher, 2024). The sample is deposited directly in the furnace, dried, chatted, and atomized in a controlled sequence. GFAAS shows detection limits 20–200 times lower than for FAAS (in the ng/L range) (Butcher, 2024), enabling precise quantification of iron in low-concentration samples such as serum or intracellular extracts. In the work by Miller-Ihli from 1989 (Miller-Ihli, 1989) GFAAS has been presented as a highly versatile method for iron analysis in biological samples. They presented different sample preparation strategies, including direct analysis of fluids, wet ashing with nitric acid and hydrogen peroxide, and dry ashing by high temperature. The method has proved to be applicable in multielement analysis in biological reference materials with high accuracy and precision, with minimal sample contamination and loss (Miller-Ihli, 1989).

### 2.3 Electrochemical methods

These methods are based on measuring the electric current or potential generated redox reactions (Lu et al., 2012). Electrochemical methods often use a working electrode that facilitates controlled deposition and oxidation/reduction of iron. Unlike spectroscopy methods, electrochemical methods do not need optical components or high temperatures. They are the most useful for detecting ultra-trace ions, real-time monitoring, and field applications (Lu et al., 2012).

#### 2.3.1 Voltammetry

Anodic Stripping Voltammetry ASV is the most widely used electrochemical technique for iron detection (Nsabimana et al., 2019; Wygant and Lambert, 2022). It has two major steps. The Fe(III) is reduced to Fe(II) or Fe^0^ and deposited on the electrode surface (Florence, 1970; Van Staden and Matoetoe, 1998; Borrill et al., 2019). Then the deposited iron is oxidized back into the solution while the current is recorded. The peak current is proportional to the iron concentration (Abollino et al., 2019; Nsabimana et al., 2019). This method offers great sensitivity that can reach low nanomolar concentrations. However, it requires careful control of electrode surface conditions, and it can be influenced by other redox reactions (Florence, 1970; Van Staden and Matoetoe, 1998; Nsabimana et al., 2019). In 1970, Florence (Florence, 1970) described the use of ASV to determine the iron by a chemical exchange between FE(III) and a bismuth-EDTA complex. Unlike earlier methods that used lead-EDTA, the bismuth EDTA complex was more stable and selective, which led to a lower detection limit (about 9 nM). This method involved deposition of bismuth onto a rotating glassy carbon electrode with a mercury film, followed by anodic stripping. The reaction has been optimized for a pH of 4 and has been used to detect iron in various water samples and chemicals (Florence, 1970). Van Staden and Matoetoe in a study from 1998 (Van Staden and Matoetoe, 1998) developed a flow-through system using differential pulse anodic stripping voltammetry (DPASV) for the detection of both Fe(II) and Fe(III). They used pyrophosphate buffer at pH 9 to stabilize the two oxidation states. Distinct peaks for Fe(III) and Fe(II) were observed at −0.8 and −0.5. They presented a detection limit of about 10 nM and an SD of less than 4%. The method showed good agreement with spectrophotometric methods (Van Staden and Matoetoe, 1998). A more recent innovation by Han et al. (2021) offers a high surface area, enhanced conductivity, and superior electrocatalytic activity toward the reduction of Fe(III). The use of a micro needle electrode sensor modified with gold nanoclusters immobilized on a conductive polymer film allows for strong reproducibility, selectivity, and a detection limit of 3.1 nM. This system avoids toxic components and does not require an additional complexing agent (Han et al., 2021).

#### 2.3.2 Amperometry

Amperometry is a technique that measures the electric current resulting from the oxidation or reduction of analytes at the surface of an electrode under controlled applied potential (Adeloju, 2004; Mross et al., 2015). When potential is applied, Fe(II) or Fe(III) undergo redox reactions at the electrode, generating a current proportional to their concentration (Adeloju, 2004; Saavedra and Cortón, 2014). Vorlíček and Vydra (1965) showed a biamperometric approach where iron (III) is titrated with EDTA using two graphite electrodes. The current drop at the endpoint serves as a precise indicator of Fe(III) concentration (Vorlíček and Vydra, 1965). Saavedra and Cortón (2014) presented a real-time amperometric sensor suitable for bacterial applications. The system’s cyclic voltammetry scans reveal two distinctive peaks: oxidation of Fe(II) and reduction of Fe(III). This dual detection enables tracking of bacterial bio-oxidation kinetics. The use of calibration curves provides a rapid and relatively interference-free quantification (Saavedra and Cortón, 2014).

#### 2.3.3 Potentiometry

Potentiometry is a classical electrochemical technique based on measuring the potential difference between a reference and an indicator electrode, and offers a versatile tool for detecting iron ions (Amos and Brown, 1963; Bralić and Radić, 1999). Amon and Brown in 1963 (Amos and Brown, 1963) established a robust titration procedure capable of determining iron (II) using potassium dichromate as an oxidizing agent (Amos and Brown, 1963). Their method leveraged a lead reductor to reduce iron (III) before titration, and the end point was precisely identified using a platinum electrode versus a saturated calomel reference. Although primarily designed for uranium-iron mixtures, the procedure shows potentiometric titration applicable to microbial samples, especially when interferences may exclude other methods (Amos and Brown, 1963). Then, a flow injection potentiometry (FIP) system for detecting Fe(III) through complexation with fluoride ions was introduced as part of advances in sensor technology (Bralić and Radić, 1999). By incorporating a fluoride ion-selective electrode into a cascade flow cell, the system achieved rapid quantification of iron (III) concentration across a wide dynamic range. The response was directly linked to the kinetics of FeF_2_^+^ complex formation. This method’s flow design makes it perfect for real-time monitoring of iron bioavailability (Bralić and Radić, 1999). Recently, a Fe(III)-selective ion electrode (ISE) was developed using piperine, an alkaloid derived from *Piper nigrum*, incorporated into a solvent polymeric membrane (Madhushani and Hasini, 2021). This approach allowed for the concentration range of 1 × 10^−4^ to 1 M in citrate buffer (pH 3.1), with a detection limit of 6 × 10^−5^ M. Analytical performance was validated against AAS, with no significant differences observed. The sensor remained functional for up to 10 weekends with minimal potential drift. Its simplicity, portability, and low cost make it a promising alternative for *in situ* iron analysis (Madhushani and Hasini, 2021).

### 2.4 Radiometric assays

Radiometric techniques are among the most precise and sensitive methods for studying iron metabolism. This method uses radioisotopes of iron, like ^55^Fe and ^59^Fe, and allow to study iron uptake, transport, and storage directly in living bacterial cells (Hantke, 1981; Lewis, 2010) These methods are typically not used to measure total iron, but for kinetic studies, which allow for precise following of iron ions in bacterial growth (Hantke, 1981). The main principle is that the iron radioisotopes are incorporated in microbial cultures in the form of ^55^FeCl_3_ or ^55^FeSO_4_. Bacteria then introduce the radiolabeled iron into their metabolism. After the incubation, the samples are centrifuged or filtered to separate the cells, and then washed to remove unbound isotope (Hantke, 1981; Lewis, 2010). Radioactivity in the pellet is measured using liquid scintillation counting. One of the first works that mentions this method was written by Hantke in 1981 (Hantke, 1981) who used ^55^Fe uptake assays to investigate the regulation of iron transport in *E. coli*. The author’s work showed how mutations in the Fur (Ferric uptake regulator) system affect iron acquisition, laying the base for further iron regulatory studies in the future (Hantke, 1981). In studies of *Porphyromonas gingivalis* (Lewis, 2010) radioisotopes were applied to measure binding affinities, uptake rates, and competition between siderophores and host iron sources (hemin). Microbial iron uptake was quantified using radioactivity assays. This allowed for the quantification of iron acquisition under various conditions (Neilands, 1981). In recent years, due to stricter safety regulations and the development of alternative methods (ICP-MS, fluorescent probes), radiometric techniques remain an essential tool in the studies of iron transport, especially where other methods lack the required sensitivity.

### 2.5 Fluorescence and chemiluminescence

Fluorescence and chemiluminescence methods offer powerful alternatives to traditional colorimetric and radiometric assays for detecting and monitoring iron in microbiological systems. These methods rely on probes or luminescent reactions that respond to iron ions.

Calcein and ester derivatives (Calcein-AM) are widely used to detect iron. Calcein is a green-fluorescent dye whose emission is quenched by Fe(II) and Fe(III) (Glickstein et al., 2005). Typically, Calcein-AM permeates microbial cells and is hydrolyzed intracellularly to Calcein, which can be quenched by iron. Chelators such as deferoxamine are used post-staining to determine the maximal fluorescence to infer iron content by difference (Glickstein et al., 2005).

Phen Green SK is another iron-sensitive dye, which exhibits fluorescence quenching in the presence of Fe(II) and Fe(III) (Petrat et al., 2000). It is useful for the determination of intracellular Fe content (Petrat et al., 1999; Petrat et al., 2000). Due to its different spectral properties compared to Calcein, it is particularly advantageous in bacterial species with high autofluorescence or when co-staining is required (Petrat et al., 1999; Oter et al., 2007; Grubwieser et al., 2024). In *Pseudomonas* species, pyoverdine is a naturally fluorescent siderophore. Its fluorescence diminishes upon iron binding. This can be used to monitor iron uptake by siderophore (Meyer, 2000).

More recently, oxidation-state-specific fluorescent probes have been developed to target either Fe(II) or Fe(III) with high selectivity (Gonciarz and Renslo, 2021). FerroOrange (RhoNox-4) is a well-characterized probe specific for cytosolic Fe(II), activated in reduction-based mechanisms in which dialkylarylamine N-oxide is selectively deoxygenized by ferrous to generate various fluorescent probes. These provide redox-specific insights and is especially useful in studies of labile Fe(II) pools in live cells and microbial cultures (Yang et al., 2022; Fu et al., 2023; Grubwieser et al., 2024). The RhoNox-family probes, including HMRhoNox-M and HMRhoNox-II, exhibit strong fluorescence enhancement upon binding to labile ferrous ions, without interference from Fe(III) or other physiologically relevant metal species (Aron et al., 2018; Gan et al., 2021). Their red fluorescence emission profile allows for easy spectral separation from commonly used green-emitting dyes such as calcein, enabling multiparameter imaging approaches. Detection limits typically reach the low micromolar range (∼0.2–1 µM). Further advancements in N-oxide chemistry probes development led to synthesis of other variants: CoNox-1 (blue), FluNox-1 (green), and SiRhoNox-1 (red) (Hirayama et al., 2017). With sub-micromolar detection limits and good membrane permeability, RhoNox probes facilitate accurate monitoring of intracellular Fe(II) dynamics and have already been employed to investigate iron-dependent virulence, stress adaptation, and antimicrobial responses in both microbial and host-pathogen systems.

In contrast, turn-off probes specialised for Fe(III) detection have been employed, such as the boron-dipyrromethene-based fluorescent probe (BODIPY-CL) (Leng et al., 2022) and the chitosan-based tetraphenylethylene (CS-TPE) fluorochrome (Wang et al., 2024). These sensors exhibit fluorescence quenching upon binding ferric iron and display exceptional selectivity over other metal ions. The BODIPY based sensor exhibited a fluorescence quenching response that was linear with Fe(III) concentrations between 0 and 400 μM, with a detection limit up to 3 μM (Leng et al., 2022). While, the sensitivity of CS-TPE was reported at ∼1 μM and showed good detection range that allows quantification of 10–300 μM of iron trace (Wang et al., 2024). Importantly, these probes retain stability in the presence of interfering agents such as phosphate or ascorbate. In complex biological systems, such probes allow direct visualization of Fe(III) distribution in single cell resolution.

Chemiluminescent iron assays are less common but also offer high sensitivity, especially for low-iron samples. These assays generate light when iron participates in a redox reaction that produces reactive oxygen species, which then react with a luminescent substrate (Pietrzak and Denes, 1996). In the luminol assay, in which luminol undergoes oxidation by H_2_O_2_ in the presence of catalytic iron, leading to emission of blue chemiluminescence. It is not iron-specific; this method is valuable when coupled with iron-specific chelators or in controlled conditions where iron is the main catalyst (Pietrzak and Denes, 1996; Khan et al., 2014).

### 2.6 Chromatographic and coupled methods

Chromatographic methods provide a way to separate and identify iron before quantification. While techniques like AAS and ICP-MS can measure total iron, chromatography allows for to resolution of specific forms of iron before detection.

#### 2.6.1 High performance liquid chromatography

High Performance Liquid Chromatography (HPLC) is used to separate iron-containing compounds, such as siderophores, iron-bound metabolites, or metalloproteins. Detection can then be performed by using variety of spectroscopic methods such as Ultraviolet-visible (UV-Vis) spectroscopy, Inductively Coupled Plasma Mass Spectrometry (ICP-MS), Inductively Coupled Plasma Optical Emission Spectroscopy (ICP-OES), or electrochemical detection. HPLC methods often involve reversed-phase or ion-exchange columns, with chelators like EDTA to maintain iron in soluble, detectable forms (Proch and Niedzielski, 2021). Work by Şenyuva from 2002 (Şenyuva et al., 2002) described the use of HPLC in post-column derivatization to separate Fe(II) and Fe(III). After separation, the reaction with the use of PAR (4-(2-pyridylazo)resorcinol) led to the creation of complexes that could be detected at 521 nm. This achieved great sensitivity (0.109 ug/L for Fe(II), 0.217 ug/L for Fe(III)) and reproducibility. It requires additional reagents and reaction time, but it is very useful for trace metal detection. The methods showed strong correlations with AAS total iron results (Şenyuva et al., 2002). Proch and Niedzielski (2021) have described the use of HPLC coupled with MIP OES (Microwave-Induced Plasma Optical Emission Spectrometry) and ICP OES (Inductively Coupled Plasma Optical Emission Spectrometry), which enabled the separation and detection of iron without a post-column procedure. Using a cation-exchange column and PDCA-cased eluent, Fe(II) and Fe(III) were resolved in under 5 min. MIP OES is a relatively new approach, using a nitrogen plasma, which demonstrates advantages in cost and simplicity, but it has higher detection limits (∼100 ug/L) than ICP OES (∼6 ug/L). MIP OES still achieved a useful detection limit and showed potential as a green alternative, given that it uses less gas (Proch and Niedzielski, 2021). High-performance liquid chromatography coupled with electrospray ionization mass spectrometry (HPLC-ESI-MS) has been used to detect hydroxamate siderophores (McCormack et al., 2003). This method involved using polystyrene-divinylbenzene stationary phase and gradient elution with methanol and formic acid, which allowed for the separation of both iron complexes and free ligands. The detection limit was very low, at about 0.23 nM for ferrioxamine. This showed that using HPLC-ESI-MS could overcome limitations of traditional assays by its sensitivity and selectivity (McCormack et al., 2003). HPLC coupled with inductively coupled plasma mass spectrometry (ICP-MS) enables direct detection of iron within organic complexes, offering a robust approach to identify and quantify iron ligands in bacterial cultures (Boiteau et al., 2013). To improve the sensitivity, the authors used iron-FREE HPLC system and minimized interference in ICP-MS with a hexapole collision cell and oxygen in the carrier gas. The method detected iron complexes from cyanobacteria and marine samples, showing its potential in tracking siderophore production and iron in natural environments (Boiteau et al., 2013).

#### 2.6.2 Ion chromatography (IC)

IC allows for the separation of ionic iron species, It is useful in studying iron redox dynamics or the release of iron from siderophores and binding proteins. It is less common than HPLC, but IC can provide insights into environmental samples and support studies of iron metabolism mutants (Schnell et al., 1998; Şenyuva et al., 2002; Michalski, 2009). The study by Schnell et al (1998), about the metabolic pattern of sulfate-reducing bacteria, Fe(III) and Fe(II) are separated on a polymer-coated silica-based cation exchange column, followed by post-column derivatization with 4-(2-pyridylazo)resorcinol (PAR) for spectroscopic detection at 520 nm (Lebel and Fu Yen, 1984; Schnell et al., 1998). The major advantage of IC is the simultaneous determination of Fe (III) and Fe(II) in bacterial cultures and environmental samples. Schnell et al. (1998) Demonstrated that reduction of Fe(III) by the *Geobacter metallireducens* can be effectively tracked and determined. Their method proved to be effective with good reproducibility and sensitivity, detecting iron at micromolar concentrations with minimal interference from other components (Schnell et al., 1998). One challenge noted by the author is the potential oxidation of Fe (II) during the process. Strategies such as conditioning columns with ascorbic acid and maintaining anoxic conditions for reagents and eluents are the key to ensuring accurate results (Schnell et al., 1998). IC can also be used to remove Fe from media to study iron-deprived bacteria and the expression of iron-regulated membrane proteins (Kadurugamuwa et al., 1987).

### 2.7 Siderophore assays (CAS and derivatives)

In microbiology, siderophore production assays are an essential tool for studying how bacteria acquire iron from the environment (Louden et al., 2011). Siderophores are small, high-affinity iron chelating compounds secreted by bacteria and fungi under iron-limited conditions (Hider and Kong, 2010; Gomes et al., 2024). While these assays do not measure iron concentration directly, they quantify the ability of microbes to bind the iron, which is a crucial part of iron metabolism in bacteria (Himpsl and Mobley, 2019). The chrome Azurol S (CAS) assay was introduced in the 1980s, is the gold standard for siderophore detection, and remains widely used today (Neilands, 1981; Himpsl and Mobley, 2019), often with minor modifications to adapt it to different sample types or screening formats. The CAS assay is based on color change. A complex of Chrome Azurol S, Fe(III), and hexadecyltrimethylammonium bromide (HDTMA) forms a stable blue complex (Louden et al., 2011). When a siderophore chelates Fe(III) from this complex, the color changes from blue to orange, which can be quantified spectrophotometrically (Himpsl and Mobley, 2019). The original CAS assay was introduced in 1987 by Neilands (1981). Initially designed as a universal way of detecting siderophores. In 1994 Payne (1994) described how this assay has become a gold standard due to its simplicity and broad application. The CAS works irrespective of the siderophore type. Payn also described both agar and liquid versions of this assay, proving its effectiveness in both quantitative and qualitative studies (Payne, 1994). By 1990, most of the assay’s limitations had been discovered. Alexander and Zuberer in 1991 (Alexander and Zuberer, 1991) highlighted that CAS was successful at identifying bacteria that produce siderophores in high quantities, but many bacteria did not grow on CAS agar or did not create a halo despite producing siderophores in liquid media. To address this problem, they modified the assays to improve their stability and adopted microtiter methods, which allowed for high-throughput siderophore quantification (Arora and Verma, 2017). Now, it can be used for quantification of siderophores by any bacteria as a better alternative to the routine colorimetric method. The understanding of siderophores itself also improves over time. Hider and Kong reviewed the chemistry of siderophores, emphasizing their high specificity for ferric iron and highlighting their relevance across a broad range of biological systems, from microbial physiology to therapeutic applications. They also elaborated on the topic of kinetic parameters that lay under siderophore and iron interactions, showing that the CAS assay remains relevant for assessing iron-binding (Hider and Kong, 2010). Building on that, recent studies have introduced a series of modifications to enhance sensitivity, specificity, and microbial compatibility of the assay. Another improvement was the development of buffer-free CAS (bf-CAS) system combined with a diluted R2A medium, allowing for the detection of siderophores in microorganisms that struggle in iron-limited media (Murakami et al., 2024). A correction factor was also introduced to account for pH-related absorbance shifts (Murakami et al., 2024). Similarly, Gomes et al., in 2024 (Gomes et al., 2024) review modifications of CAS assay that addressed limitations such as toxicity of HDTMA by substituting it with less harmful alternatives like DDAPS (N-Dodecyl-N,N-dimethyl-3-ammonio-1-propanesulfonate), or by using overlay and double-layered agar to enhance microbial growth. They also described the shift toward alternative dyes and the importance of the microplate approach that reduces reagent use and increases screening throughput (Gomes et al., 2024).

The CAS assay has evolved from a simple chemical tool to a widely used standard method for studying iron metabolism. Enhanced by complementary techniques to distinguish between specific siderophore types, it remains an essential tool for investigating microbial iron acquisition and potential antimicrobial strategies (Ferreira et al., 2019; Osman et al., 2019).

### 2.8 Molecular biology method for studying iron regulation in bacteria

While chemical methods quantify iron, molecular biology tools are crucial for understanding how bacteria sense, regulate, and respond to iron availability. The bacterial regulation system is focused around the Fe(III) uptake regulator (Fur), siderophore biosynthesis, and iron transport genes. In this section, we will focus on describing methods that have been used to study these processes in bacteria.

#### 2.8.1 Reporter gene assays

Reporter gene assays are fundamental tools for studying the regulation of iron-responsive genes in bacteria (Escolar et al., 1999; Jiang et al., 2008). These assays rely on cloning an iron-regulated promoter upstream of a reporter gene, allowing for assessing promoter activity in response to iron availability or genetic modifications (Cotter et al., 1992; Escolar et al., 1999; New et al., 2003). Common reporter systems include: LacZ (β-Galactosidase), GFP (green fluorescent protein), Lux (bacterial luciferase), and *luc* (firefly luciferase) (New et al., 2003). These reporters produce measurable outputs that reflect the activity of the iron-responsive promoter, offering a nondestructive and real-time readout. The general procedure consists of cloning iron-regulated gene (Cotter et al., 1992; New et al., 2003) (e.g., *fur, feoB*, siderophore synthesis genes) upstream of the reporter gene, then the recombinant plasmid is transformed into bacteria, bacteria are grown, and the activity of the reporter is measured (Braun and Burkhardt, 1982; Cotter et al., 1992; Dancis et al., 1992; Escolar et al., 1999). In *E. coli,* the *P_fur::lacZ* fusion has been widely used to monitor Fur-dependent repression (Escolar et al., 1999). These fusions have been crucial for discovering new members of the Fur regulon, broadening the understanding of iron global impact of bacterial physiology (Escolar et al., 1999). A lacZ fusion approach has been used to investigate how iron availability affects respiratory gene expression in *Escherichia coli*. Under iron-limiting conditions, anaerobic respiration genes were selectively downregulated, while aerobic respiration systems showed slight upregulation, revealing additional layers of iron-responsive regulation beyond the canonical Fur pathway (Cotter et al., 1992). Overall, the reporter systems are a very versatile methodology that can be fused to any promoter of interest. Advances in genetic manipulation techniques in recent years have improved their accessibility and made it easier to introduce targeted mutations, even in clinical strains (Strzelecki et al., 2024).

#### 2.8.2 Transcriptional studies

qPCR and RNA-seq are important tools for measuring the expression of iron-regulated genes at the transcriptional level. These techniques enable precise, sensitive detection of mRNA transcripts, allowing for identification of changes in gene expression in response to iron availability or genetic modifications.

Quantitative PCR can be used to detect mRNA levels by amplifying reverse-transcribed cDNA using sequence-specific primers and monitoring the accumulation of PCR product in real time. To study the regulation of iron, bacteria are grown under iron-limited conditions. Total RNA is extracted, converted to cDNA, and iron-regulated genes are quantified (Rocha et al., 2020). The study by Nilsen and Boye in 2005 (Nielsen and Boye, 2005) used this method to analyze gene expression in *Actinobacillus pleuropneumoniae* under iron-depleted conditions. The authors focused on identifying suitable housekeeping genes for iron studies. They demonstrated significant upregulation of *tbpA, exbB*, and *fhuD* genes under iron-limited conditions, confirming their role in iron metabolism (Nielsen and Boye, 2005). Also, the regulatory networks governing iron transport and homeostasis in soil related *Pseudomonas fluorescens* have been investigated through genes expression analysis (Fortuna et al., 2019). Bacteria were exposed to nanoscale zero-valent iron, and the expression of *pvdS* (regulator of siderophore pyoverdine synthesis) and bacterioferritin-associated ferredoxin gene (involved in iron storage). The qPCR results were cross-validated with culture-based methods. This work highlighted the importance of these methods in confirming whether tested compounds alter the gene expression of iron-related genes (Fortuna et al., 2019). Quantitative PCR is a sensitive, reproducible, and versatile method for studying bacterial iron acquisition and homeostasis. Whether investigating virulence in pathogens or ecological responses in environmental isolates, qPCR provides high-resolution insight into bacterial adaptation to iron availability. By following best practices such as primer design, reference gene validation, and cross-method verification, researchers can use qPCR to advance our understanding of bacterial iron metabolism.

RNA-seq involves high-throughput sequencing of cDNA libraries prepared from total bacterial RNA. These methods provide global gene expression profiles, identification of novel iron-regulated genes and non-coding RNAs involved in iron metabolism (Butcher and Stintzi, 2013; Liu et al., 2017; Ibraim et al., 2019). Transcriptomics has been applied to investigate gene expression changes in *Riemerella anatipestifer* under iron-limited conditions (Liu et al., 2017). By culturing bacteria with and without an iron chelator, they identified 463 genes: 80 upregulated (mainly involved in iron acquisition) and 383 downregulated (Liu et al., 2017). Similar experiments have also been done by other authors linking iron regulation to bacterial motility, and discovering non-coding RNAs, many of which were iron or Fur-regulated, suggesting RNA-based regulatory mechanisms in iron homeostasis (Butcher and Stintzi, 2013). The results highlight iron’s central role in bacterial metabolism and demonstrate RNA-seq’s role in expanding the knowledge about iron’s role in biological systems.

## 3 Discussion

Despite the availability of a wide range of analytical tools, evaluating iron in microbial samples remains methodologically challenging. Each technique presents specific limitations that influence accuracy, sensitivity, and applicability under various experimental conditions. The advantages and limits of described approaches were summarized in Table 2.

**TABLE 2:** Comparative overview of analytical methods for iron detection in biological and environmental samples.

Method type	Advantages	Limitations
Colorimetric assays	Widely accessible and cost-effective; suitable for routine screening in microbiology and environmental studies. Broad effective working pH range (pH 3.0–9.0). Evaluation of non-protein-bound iron in plasma, cerebrospinal fluid, or microdialysates. Can also be used indirectly to monitor iron binding in siderophore assays or oxidation assays.	Limited sensitivity and selectivity; prone to interference from other metal ions.
Atomic absorption spectroscopy	High accuracy for total iron. Depending on technique choice enable high-throughput or precise analysis for low-concentration samples (ppb range).	Requires sample digestion, which can be time-consuming and may lead to loss or alteration of iron species; Flame AAS has moderate sensitivity, which may be insufficient for trace iron detection in some microbial or environmental samples; Graphite Furnace AAS improves sensitivity but is slower and requires more specialized equipment and expertise.
Electrochemical methods	High sensitivity; allows real-time detection of trace iron in various matrices.	Susceptible to interference from other redox-active species; requires careful calibration.
Radiometric assays	Exceptional sensitivity and specificity for studying iron uptake and metabolism.	Involve handling radioactive materials, which require specialized facilities and safety protocols. Not suitable for routine or high-throughput analysis due to regulatory and practical constraints.
Fluorescence and chemiluminescence methods	Enables intracellular or *in situ* detection; rapid and adaptable to various biological contexts. Useful in oxidation-related studies Allows differentiation of Fe(II)/Fe(III).	May be affected by autofluorescence or indirect detection; requires appropriate controls. Chemiluminescence assays are indirect and often measure ROS related to iron catalysis rather than iron itself.
Chromatography	Detailed speciation and separation of iron species. HPLC is suitable for siderophores, iron complexes (e.g., heme) in serum or microbial extracts. IC enabling redox studies, analysis of iron speciation in environmental or clinical samples.	Require careful sample handling to prevent oxidation or alteration of iron states during analysis.
Siderophore assays	Gold standard for siderophore based iron transport studies in cell cultures or animal models. Useful for screening.	Specific to siderophore mediated Fe acquisition systems activity and do not provide information on other iron pools or species.
Molecular biology	Provides insight into iron-regulated gene expression and cellular responses. Identifies both known and novel iron-regulated genes microbial responses to iron limitation/overload.	Indirect measure of iron status; results may be influenced by multiple regulatory factors.

Colorimetric assays primarily detect ferrous iron Fe(II). Since iron in biological systems often exists in the ferric form Fe(III), which introduces additional sample handling, potential variability, and a risk of analytical artifacts. These assays are also prone to interference from other metal ions or sample matrix components, requiring the use of masking agents or tightly controlled conditions (Table 2). Sensitivity remains a challenge, particularly at low iron concentrations or in complex biological matrices where extensive sample preparation may be necessary (Giacalone et al., 2025). While widely used as a standard approach to evaluate siderophore production, these assays only provide indirect information about iron status and do not quantify total iron (Louden et al., 2011). Furthermore, they are limited to detecting siderophore-mediated iron acquisition and offer no insight into other iron pools or redox states (Louden et al., 2011; Murakami et al., 2024).

Atomic level studies like AAS is a widely accepted and selective technique for quantifying total iron, but it requires digestion of biological samples (Putri Maharani et al., n.d.). This step is not only time-consuming but may also lead to the loss or alteration of specific iron species. Flame AAS, though accessible, offers moderate sensitivity that may be inadequate for detecting trace iron levels. Graphite furnace AAS improves sensitivity but involves slower throughput and demands more advanced instrumentation and user expertise (Putri Maharani et al., n.d.; Miller-Ihli, 1989).

Electrochemical methods provide high sensitivity and the advantage of real-time detection. However, they require careful electrode preparation, regular calibration, and are susceptible to interference from redox-active compounds in complex biological samples (Florence, 1970; Liu et al., 2017). Potentiometry, although historically significant, has limited application in modern speciation studies unless coupled with other techniques (Amos and Brown, 1963; Bralić and Radić, 1999).

Fluorescence-based probes enable fine intracellular iron detection and are easy to handle, but face limitations related to autofluorescence and require equipment accessibility (Petrat et al., 1999; Petrat et al., 2000). Radiometric assays provide exceptional specificity but are impractical for routine use due to regulatory and safety requirements (Hantke, 1981; Lewis, 2010). Chromatographic methods provide detailed speciation enable separation and quantification of specific iron species, but require advanced instrumentation and expertise. The complexity and cost of instrumentation, along with the need for technical expertise and post-column detection systems, make them less accessible for routine analysis (Proch and Niedzielski, 2021).

**FIGURE 2:**
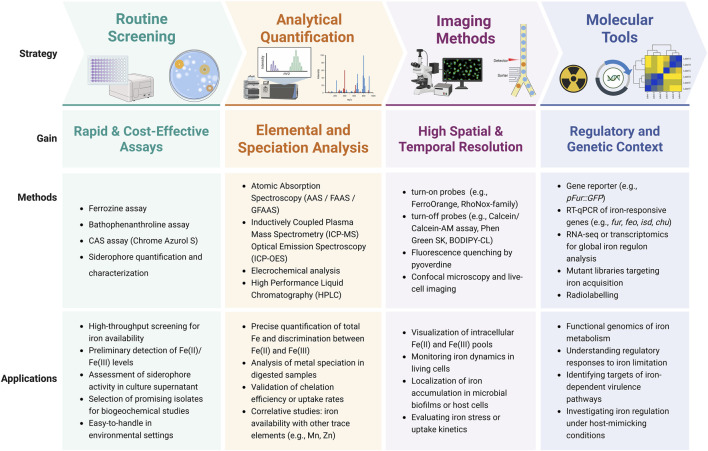
Methodological strategies to explore microbial iron homeostasis. The figure summarizes four methodological levels: routine screening assays for rapid and cost-effective detection, analytical quantification for precise elemental and speciation analysis, imaging methods for high spatial and temporal resolution in live cells and biofilms, and molecular tools providing regulatory and genetic context. Representative techniques and typical applications are indicated for each level.

Molecular biology methods, give deeper insight on iron biological functions toward transcriptional profiling, provide valuable information on iron-regulated gene and small RNAs expression (Chareyre and Mandin, 2018). However, these approaches are indirect and do not measure iron concentration itself. Their results may be influenced by additional regulatory factors, reducing their utility for precise quantification of iron dynamics. Nevertheless, while numerous methods are available for investigating microbial iron biology, each has specific limitations and advantages. Careful consideration of the biological question, required sensitivity, sample type, and technical resources is essential for selecting the most appropriate method or combination of methods for comprehensive iron analysis. Practical workflow of using complementary technologies was presented on scheme (Figure 2).

## 4 Perspectives

The field of iron detection and regulation in microbes (coupling bacteria, fungi and bacteriophages) is rapidly evolving, driven by the need for better sensitivity, specificity, and real-time monitoring of iron in biological systems. While classical techniques such as colorimetric assays have laid a solid foundation, emerging methods are expanding both the technical capabilities and biological insight available to researchers.

Several key trends and future directions are shaping this area:

• High-Throughput and Automated Screening–The development of minimalized, automated platforms is enabling high-throughput screening of microbial mutant strains, environmental isolates, and small molecules that influence iron uptake. Microfluidic systems and robotic liquid handlers are increasingly integrated with colorimetric, fluorescence, and luminescence-based assays, allowing for rapid testing of iron acquisition phenotypes (Weber et al., 2024;Ceriotti et al., 2025).
• Single cell and spatially resolved techniques–A growing focus has also recently been placed on looking directly into single cells and spatial mapping of iron within bacterial populations and biofilms. Techniques such as X-ray fluorescence microscopy, Nanoscale secondary ion mass spectrometry, Fluorescence-lifetime imaging microscopy (De Jonge and Vogt, 2010; Miller and Ralle, 2024). This can help explain how iron distribution varies across individual cells, advancing the understanding of microbial systems.
• There is a significant interest and need in the design of next-generation iron probes and biosensors with better selectivity (between Fe(II) and Fe(III)), sensitivity (detection of lower concentrations), and biocompatibility for live-cells and *in vivo* studies (Nosrati et al., 2018). Moreover, natural compounds can act as iron chelators, helping to sense or remove excess iron from studied specimen (Karczewska et al., 2024; Taveepanich et al., 2024; Strzelecki et al., 2025). This approach can lead to an increase in the potential of new sensors or factors with anti-viral properties.
• Future research integrates iron quantification and regulation studies with multi-omics approaches combining transcriptomic, proteomics, metabolomic, and metallomics. The system biology approach is very important to further understand iron homeostasis and its cross-regulation with other components and cycles that are present in the cell (Miethke and Marahiel, 2007).
• There is also increased interest in sustainable and green chemistry approaches that minimize the use of hazardous reagents and promote the creation of environmentally sustainable iron detection kits.
• The application of novel, tractable host-pathogen models is expanding to support high-throughput studies of iron dynamics in co-culture systems and microbiomes. These models are expected to accelerate the discovery of new therapeutic strategies (Consentino et al., 2021; Abugessaisa et al., 2022; Budziaszek et al., 2023).
• Artificial intelligence and machine learning are beginning to impact the field by optimizing assay conditions, predicting iron binding motifs and regulatory elements in genomes, and interpreting complex data sets from iron-related experiments. *In silico* models are a complement to experimental research and can fill gaps in scientific knowledge.

## 5 Conclusion

This review highlights the diversity and evolution of iron detection methods applicable to microbiological research. While the reviewed methods cover a broad range of analytical approaches for assessing iron in microbial samples, each has inherent limitations related to sensitivity, specificity, sample preparation complexity, interference, and applicability to different sample types. No single method provides a comprehensive solution; therefore, selecting appropriate techniques based on the research question and sample characteristics is critical. Combining complementary methods may be necessary to overcome individual limitations and achieve accurate and reliable assessment of microbial iron dynamics. From classic colorimetric assays to advanced methods of molecular biology, each method offers a unique advantage for quantifying iron and assessing its biological importance. The continuous development and improvement of these methodologies, especially the advancement in sensitivity, real-time, species-specific tools, enhances the ability to study bacterial iron metabolism with precision. Further research should focus on optimization of these approaches, particularly in the complex and environmentally relevant samples, to deepen our understanding of iron role of microbial metabolism and pathogenesis.
